# Electrical cardiometry for non-invasive cardiac output monitoring: a method comparison study in patients after coronary artery bypass graft surgery

**DOI:** 10.1007/s10877-024-01246-y

**Published:** 2024-12-11

**Authors:** Gillis Greiwe, Rami Saad, Alexander Hapfelmeier, Niklas Neumann, Pischtaz Tariparast, Bernd Saugel, Moritz Flick

**Affiliations:** 1 Department of Anesthesiology, Center of Anesthesiology and Intensive Care Medicine, University Medical Center Hamburg-Eppendorf, Martinistrasse 52, 20246 Hamburg, Germany; 2 Institute of AI and Informatics in Medicine, TUM School of Medicine and Health, Technical University of Munich, Munich, Germany; 3 Department of Intensive Care Medicine, Center of Anesthesiology and Intensive Care Medicine, University Medical Center Hamburg-Eppendorf, Hamburg, Germany; 4 Outcomes Research Consortium, Cleveland, OH USA

**Keywords:** Pulmonary artery catheter, Pulmonary artery thermodilution, Hemodynamic monitoring, Thoracic electrical bioimpedance, Electrical cardiometry, Cardiac output

## Abstract

Cardiac output can be estimated non-invasively by electrical cardiometry with the ICON® monitor (Osypka Medical GmbH, Berlin, Germany). Conflicting results have been reported regarding the cardiac output measurement performance of electrical cardiometry. In this prospective method comparison study, we compared cardiac output measured using electrical cardiometry (EC-CO; test method) with cardiac output measured using intermittent pulmonary artery thermodilution (PATD-CO; reference method) in patients after coronary artery bypass graft (CABG) surgery. We calculated the mean of the differences with 95%-limits of agreement (95%-LOA) and their corresponding 95%-confidence intervals (95%-CI) using Bland–Altman analysis and calculated the percentage error. We also analyzed trending using four-quadrant plot analysis. We analyzed 157 paired cardiac output measurements of 41 patients. Mean ± standard deviation PATD-CO was 5.1 ± 1.3 L/min and mean EC-CO was 5.3 ± 1.3 L/min. The mean of the differences ± SD between PATD-CO and EC-CO was −0.2 (95%-CI −0.5 to 0.2) ± 1.2 L/min with a lower 95%-LOA of −2.6 (95%-CI −3.1 to −2.0) L/min and an upper 95%-LOA of 2.3 (95%-CI 1.6 to 2.9) L/min. The percentage error was 47% (95%-CI, 37 to 56%). The concordance rate for cardiac output changes was 48%. In this study, the agreement between EC-CO and PATD-CO was not clinically acceptable in patients after CABG surgery. The trending ability of EC-CO was poor.

**Supplementary Information:**

The online version contains supplementary material available at 10.1007/s10877-024-01246-y.

## Introduction

Cardiac output is a key variable of advanced hemodynamic monitoring and management in high risk surgical and critically ill patients [1]. Cardiac output monitoring can be invasive, minimally invasive, or non-invasive [2, 3]. The clinical gold standard for cardiac output monitoring is intermittent pulmonary artery thermodilution with a pulmonary artery catheter, but its clinical use is restricted due to its invasiveness [4].

Electrical cardiometry is a non-invasive method for continuous cardiac output monitoring based on the principle of thoracic electrical bioimpedance [5, 6]. In short, electrical cardiometry estimates stroke volume based on changes in thoracic electrical conductivity during the cardiac cycle [5, 7]. However, conflicting results have been reported regarding electrical cardiometry derived cardiac output measurement performance [8].

In this prospective method comparison study, we therefore compared cardiac output measured using electrical cardiometry (EC-CO; test method) with cardiac output measured using intermittent pulmonary artery thermodilution with a pulmonary artery catheter (PATD-CO; reference method) in patients after coronary artery bypass graft (CABG) surgery.

## Methods

### Study design

This was a prospective cardiac output method comparison study performed at the Center of Anesthesiology and Intensive Care Medicine at the University Medical Center Hamburg-Eppendorf (Hamburg, Germany) between October 2021 and November 2022. This study was approved by the ethics committee (Ethikkommission der Ärztekammer Hamburg, Hamburg, Germany) on March 16, 2020. All patients provided written informed consent.

### Patients

We included adults scheduled for elective CABG surgery in whom hemodynamic monitoring with a pulmonary artery catheter was planned for clinical reasons. We did not include patients with severe heart valve diseases, hemodynamically relevant arrhythmias, intracardiac shunts, or pulmonary hypertension. We excluded patients if we were unable to conduct the study measurements (e.g. agitated patient with excessive movement, transfer to the operating room for redo surgery, removal of the pulmonary artery catheter, impossibility to establish the skin electrodes for electrical cardiometry due to anatomic anomalies or bandaged wound).

### Study measurements

After anesthesia induction, a 7-Fr pulmonary artery catheter (Arrow; Teleflex Medical Europe, Westmeath, Ireland) was inserted and connected via pressure transducers to the patient bedside monitor. After surgery, all patients were transferred to the intensive care unit. During the study measurements, patients were either sedated and intubated or awake and spontaneously breathing. No study related interventions were performed during the measurement period. After ensuring sufficient signal quality, simultaneous EC-CO (test method) and PATD-CO (reference method)measurements were performed at four time points with at least 10 min between measurements (Supplementary Fig. 1).

We measured EC-CO using the ICON® monitor (Osypka Medical GmbH, Berlin, Germany) [5]. Electrical cardiometry is based on the principle of thoracic electrical bioimpedance and detects changes in thoracic electrical conductivity attributed to the pulsatile alignment of erythrocytes during the cardiac cycle in the ascending aorta [5–7]. These changes in conductivity are detected by iSense® skin electrodes (Osypka Medical GmbH) and correlate with changes in stroke volume [5, 7]. Details of the measurement principle of electrical cardiometry have been previously described [5, 7]. iSense® skin electrodes were placed at the base of the right neck above the sternoclavicular junction, under the right mastoid, on the left thorax in the mid-axillary line at the level of the xiphoid process, and 7 cm caudal of the latter electrode in the mid-axillary line [7]. We entered sex, age, height, and body weight into the ICON® monitor and enabled the pacemaker detection. Criteria for good signal quality were a heart rate detected by the ICON® monitor equal to the heart rate detected from the electrocardiogram, a physiological thoracic electrical bioimpedance signal waveform, and an ICON® signal quality indicator (SQI) ≥ 70% [9]. We recorded the raw bioimpedance waveforms over the time span of the corresponding PATD-CO injections. The bioimpedance waveforms were exported using the iControl™ software (Osypka Medical GmbH). EC-CO values were post hoc calculated using the current software version (ICON® 3.11.4) and averaged over the time span of the corresponding PATD-CO injections for statistical analysis.

For PATD-CO measurements, we averaged a set of five single PATD-CO measurements. For each single measurement we manually injected a bolus of 10 mL ice-cold saline solution randomly over the respiratory cycle. We visually inspected the resulting thermodilution curves for artefacts and PATD-CO measurements were recorded on the bedside patient monitor (Dräger Infinity® Delta, Dräger AG, Lübeck, Germany).

### Statistical analysis

Descriptive statistics are presented as median with range or mean ± standard deviation (SD) for continuous data and as absolute frequencies and percentages for categorical data. To assess the agreement between EC-CO and PATD-CO, we performed Bland–Altman analysis accounting for multiple observations per patient [10, 11]. We calculated the mean of the differences (reference—test method), the SD of the differences, and the 95% limits of agreement (95%-LOA) (mean difference ± 2.0 × SD of the differences) with the corresponding 95% confidence interval (95%-CI). We also calculated the percentage error (percentage error; 2.0 × SD of the differences divided by the mean cardiac output measurements) with 95%-CI [12]. All 95%-CIs were calculated by the percentile bootstrap, repeatedly drawing 5,000 samples of the data with replacement at patient level to preserve the within-patient correlation structure. We a priori defined a clinically acceptable agreement as a percentage error of 30% or less [12]. We assessed the ability of EC-CO to detect changes in cardiac output by comparing EC-CO to PATD-CO. We calculated the concordance rate based on the four-quadrant plot analysis using a central exclusion zone of 10% [13, 14]. A concordance rate of more than 92% was a priori defined as good [15].

Based on similar cardiac output method comparison studies [16, 17] and current recommendations [13], we planned to include complete datasets with 4 measurements per patient from 40 patients in the final analysis. We used R version 4.1.3 (The R Foundation for Statistical Computing, Vienna, Austria) for statistical analyses.

## Results

**Fig. 1:**
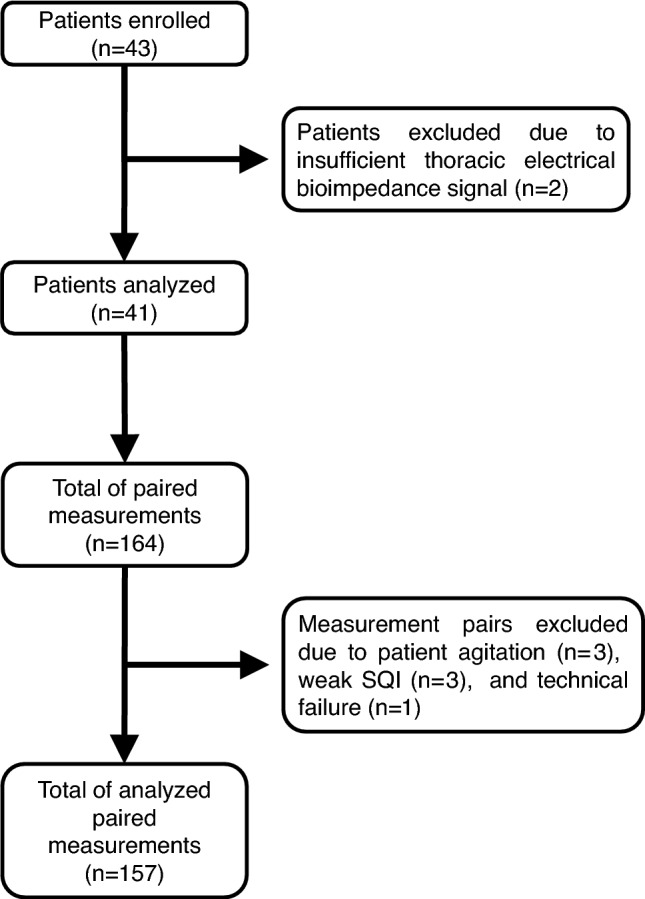
Flow chart illustrating enrollment and reasons for exclusion of patients and paired measurements. *SQI* signal quality indicator

**Fig. 2:**
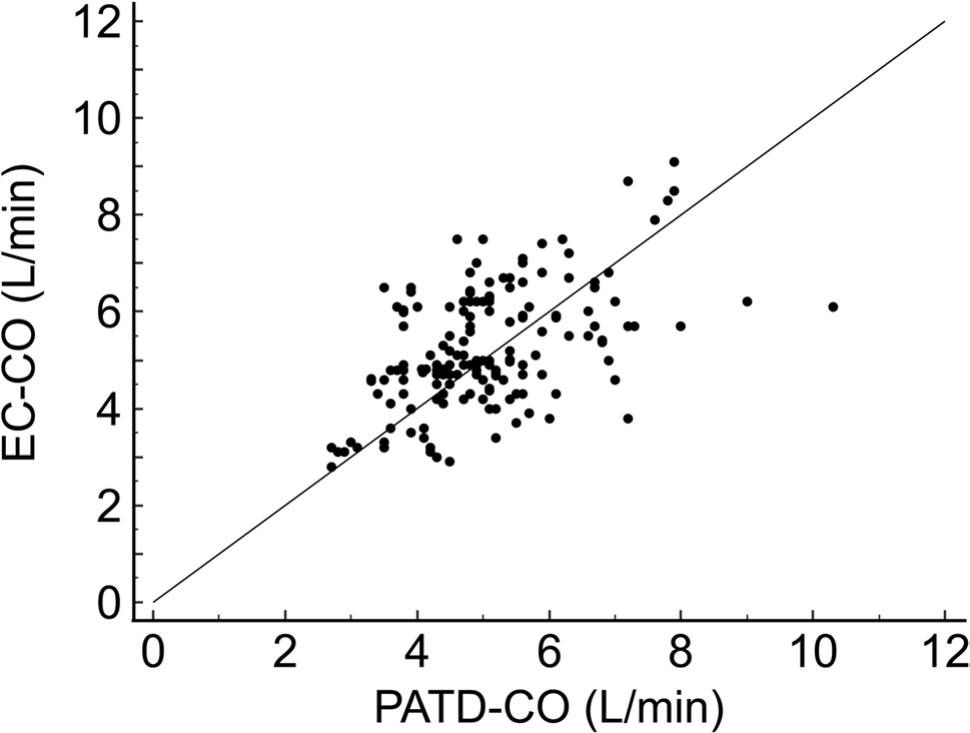
Scatter plot illustrating the relation between pulmonary artery thermodilution cardiac output (PATD-CO; reference method) and electrical cardiometry cardiac output (EC-CO; test method) measurements

We included 43 patients in this study, but could not perform EC-CO measurements in two patients because signal quality was poor. We also excluded 7 paired cardiac output measurements from 6 patients from the analysis, three because patients were agitated during measurements, three because the SQI was weak, and one because a technical failure occurred. We thus included 41 patients (Fig. 1) with a total of 157 paired EC-CO and PATD-CO measurements (Fig. 2) in the final analysis. Patient characteristics and clinical data are shown in Table 1. No adverse event occurred.

**Table 1:** Patient characteristics and clinical data

	Number of included patients (n = 41)
Intubated patients, n	28 (68%)
Age, years	67.7 ± 9
Female, n	8 (20%)
Height, m	1.75 ± 0.08
Weight, kg	84.3 ± 15.8
Body mass index, kg/m^2^	27.5 ± 4.0
History of myocardial infarction, n	15 (37%)
Diabetes mellitus, n	14 (34%)
Arterial hypertension, n	34 (83%)
Hyperlipidaemia, n	25 (61%)
Preoperative LVEF	
LVEF > 52%, n	23 (56%)
LVEF 41–52%, n	17 (42%)
LVEF < 41%, n	1 (2%)
Underlying rhythm	
Sinus rhythm, n	28 (68%)
Atrial fibrillation, n	1 (2%)
External pacing, n	12 (29%)
Vasopressor support	
Norepinephrine, n	17 (42%)

Data are presented as mean and standard deviation or absolute and relative frequencies. *LVEF* left ventricular ejection fraction

**Fig. 3:**
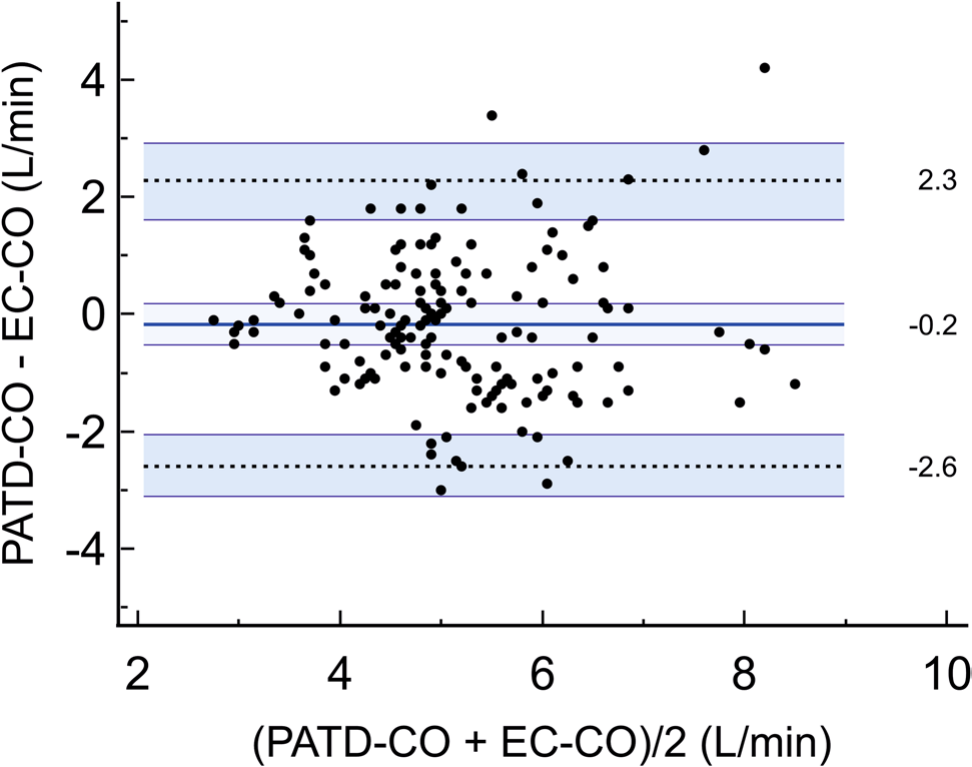
Bland–Altman plot illustrating mean of the differences (bold horizontal line) and 95% limits of agreement (upper and lower dotted horizontal lines) between pulmonary artery thermodilution cardiac output (PATD-CO; reference method) and electrical cardiometry cardiac output (EC-CO; test method). Shaded areas represent 95% CI

**Fig. 4:**
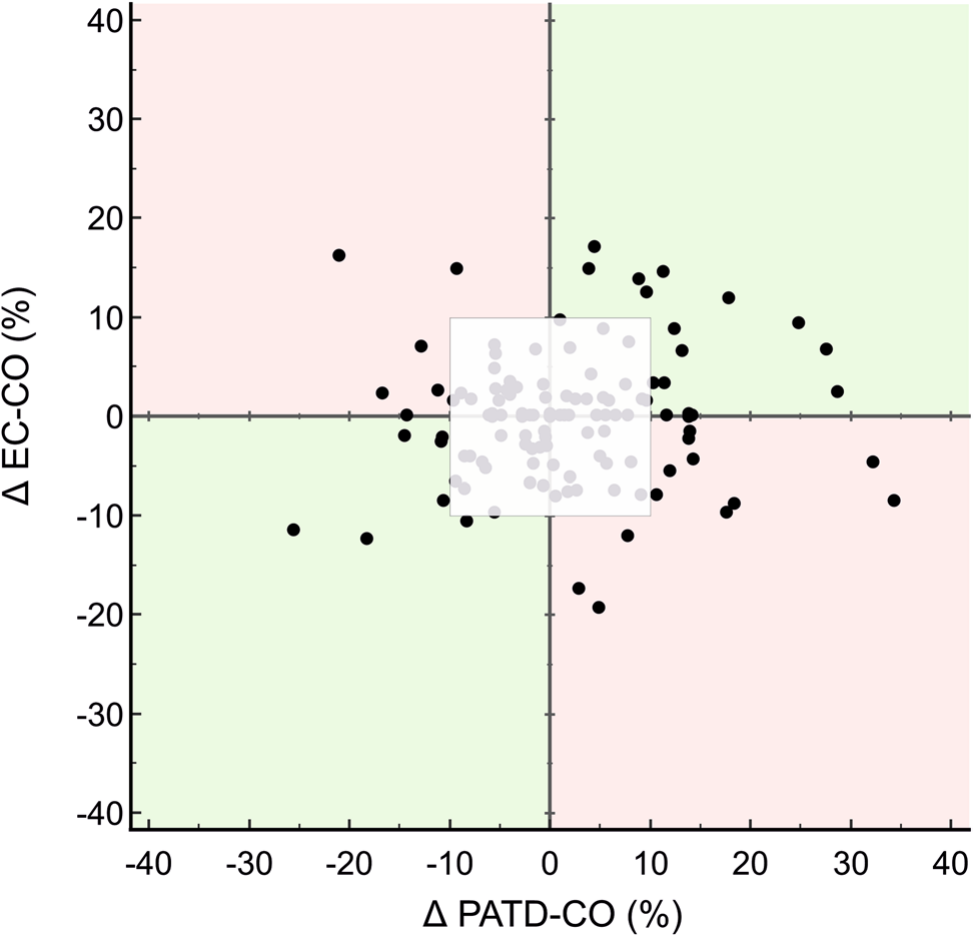
Four quadrant plot with a central exclusion zone of 10%. The concordance rate for cardiac output changes between measurement points was 48%. *PATD-CO* pulmonary artery thermodilution cardiac output, *EC-CO* electrical cardiometry cardiac output

Mean ± standard deviation PATD-CO was 5.1 ± 1.3 L/min and mean EC-CO was 5.3 ± 1.3 L/min. The mean of the differences ± SD between PATD-CO and EC-CO was −0.2 (95%-CI −0.5 to 0.2) ± 1.2 L/min with a lower 95%-LOA of −2.6 (95%-CI −3.1 to −2.0) L/min and an upper 95%-LOA of 2.3 (95%-CI 1.6 to 2.9) L/min (Fig. 3). The percentage error was 47% (95%-CI 37 to 56%). The concordance rate between changes in EC-CO and PATD-CO was 48% (Fig. 4).

## Discussion

In this prospective method comparison study, we compared EC-CO (test method) with PATD-CO (reference method) in patients after CABG surgery. The absolute agreement between EC-CO and PATD-CO was not clinically acceptable with a percentage error of 47%. The trending ability of EC-CO was poor with a concordance rate of 48%.

A meta-analysis of 13 studies assessing the cardiac output measurement performance of electrical cardiometry reported a pooled mean of the differences of 0.03 L/min with 95%-LOA from −2.78 to 2.84 L/min [8]. The mean pooled weighted percentage error was 48%, which is very similar to the percentage error in our study. However, only two of the 13 studies in the meta-analysis compared electrical cardiometry with intermittent pulmonary artery thermodilution in patients after cardiac surgery – and both studies were done more than 15 years ago [18, 19]. One of these studies included 29 patients who had CABG, valve, or combined surgeries and reported a percentage error of 70% based on 29 postoperative measurement pairs – i.e., one per patient [8, 18]. In the other study, which included 16 patients after CABG surgery, the percentage error based on 29 measurement pairs was 32% [19]. We also used intermittent pulmonary artery thermodilution as the reference method but included more patients and recorded up to 4 measurement pairs per patient.

In our study, the percentage error between EC-CO and PATD-CO was 47% and the 95%-CI ranged from 37 to 56%. This wide 95%-CI indicates that the measurement agreement between EC-CO and PATD-CO varied substantially among individual patients – although our cohort was homogenous and mostly included older men with cardiac comorbidities. The percentage error of 47% is well higher than the pre-defined 30% threshold for clinically acceptable agreement – but only slightly higher than the 45% threshold that has been proposed to define clinical interchangeability when comparing non-invasive cardiac output monitoring methods with thermodilution reference methods [20].

Measuring cardiac output with electrical cardiometry requires an accurate and reliable thoracic electrical bioimpedance signal. We thus meticulously checked the bioimpedance waveform displayed on the electrical cardiometry monitor before each measurement and verified that the SQI was above 70% to ensure proper signal quality. As we made sure that patients did not excessively move or shivered it seems unlikely that signal quality was impaired by motion artifacts. We performed our study in an intensive care unit and about one third of our patients had external pacing. Electrical artifacts thus may have influenced the electrical cardiometry measurements. It has been suggested that electrical devices in proximity to the bedside electrical cardiometry monitor may impair the thoracic bioimpedance signal [9, 18]. In a post-hoc secondary analysis, we therefore restricted our analysis to patients who did not have external pacing. This analysis yielded results similar to those of our primary analysis – with a mean of the differences ± SD between PATD-CO and EC-CO of 0.1 ± 1.1 L/min and 95%-LOA of −2.1 to 2.4 L/min (percentage error 44%). Additionally, surgical trauma with sternotomy [21] as well as the use of wire cerclages and chest tube drainages [22] may have affected our measurements. Additional factors potentially influencing the measurement performance of thoracic electrical bioimpedance measurements include incorrect positioning of skin electrodes, obesity, pleural effusions, and pulmonary edema [23–26].

Cardiac output may rapidly change due to changes of heart rate, preload, afterload, and myocardial contractility. The ability to detect cardiac output changes over time is thus essential for cardiac output monitors. We used four-quadrant plot analysis to compare the cardiac output changes detected using electric cardiometry and pulmonary artery thermodilution. The low concordance rate of 48% suggests that the trending ability of EC-CO was poor. However, most cardiac output changes were subtle as we included patients who were treated in an intensive care unit after CABG surgery and who were hemodynamically stable. We also did not perform study-related interventions to induce cardiac output changes.

This was a single center method comparison study including hemodynamically stable patients who were treated in an intensive care unit after CABG surgery. Naturally, the results are therefore not be generalizable to other settings, e.g. hemodynamically instable patients with rapid changes in cardiac output. Patient recruitment for this study was based on the availability of investigators, which may have caused a selection bias.

## Conclusion

In this study, the agreement between EC-CO and PATD-CO was not clinically acceptable in patients after CABG surgery. The trending ability of EC-CO was poor. Clinicians should not use electrical cardiometry to measure cardiac output in patients after CABG surgery.

## Supplementary Information

Below is the link to the electronic supplementary material.

Supplementary file1 (PDF 17 KB)

## Data Availability

No datasets were generated or analysed during the current study.
