# Supplementary material for: Electrical cardiometry for non-invasive cardiac output monitoring: a method comparison study in patients after coronary artery bypass graft surgery
Source: J Clin Monit Comput. 2024 Dec 11;39(2):371–6. doi: 10.1007/s10877-024-01246-y (PMC12049288; doi:10.1007/s10877-024-01246-y)
Supplement: Supplementary file 1 — Supplementary file1 (PDF 17 KB) [file 10877_2024_1246_MOESM1_ESM.pdf]

Supplementary Figure 1

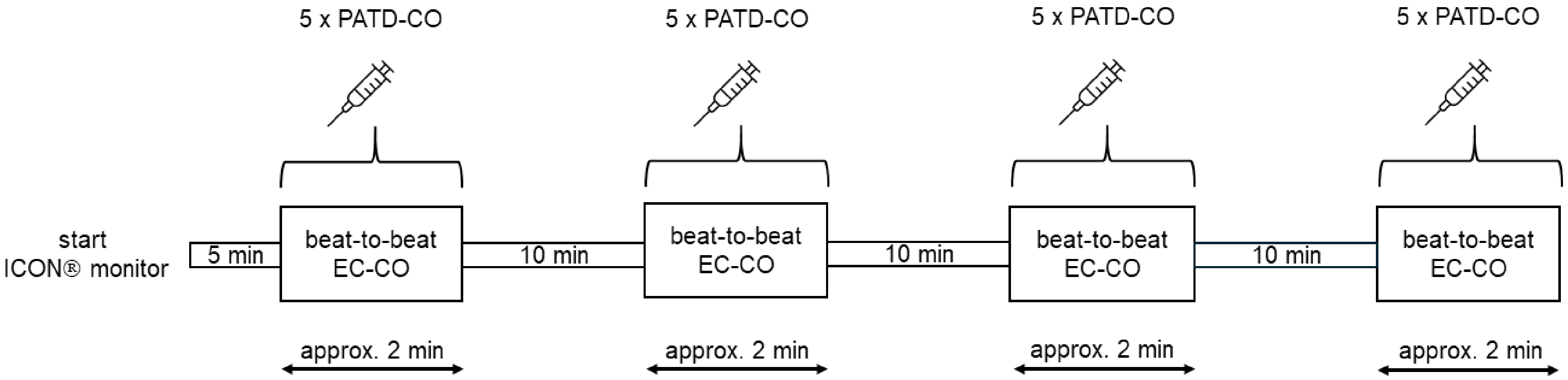

Schematic overview of the study measurements with simultaneous recording of pulmonary artery thermodilution cardiac output (PATD-CO) and electrical cardiometry cardiac output (EC-CO).
